# An Automated Micro-Total Immunoassay System for Measuring Cancer-Associated α2,3-linked Sialyl *N*-Glycan-Carrying Prostate-Specific Antigen May Improve the Accuracy of Prostate Cancer Diagnosis

**DOI:** 10.3390/ijms18020470

**Published:** 2017-02-22

**Authors:** Tomokazu Ishikawa, Tohru Yoneyama, Yuki Tobisawa, Shingo Hatakeyama, Tatsuo Kurosawa, Kenji Nakamura, Shintaro Narita, Koji Mitsuzuka, Wilhelmina Duivenvoorden, Jehonathan H. Pinthus, Yasuhiro Hashimoto, Takuya Koie, Tomonori Habuchi, Yoichi Arai, Chikara Ohyama

**Affiliations:** 1 Department of Urology, Hirosaki University Graduate School of Medicine, Hirosaki 036-8562, Japan; ishikawa.tomokazu@wako-chem.co.jp (T.I.); tobisawa@hirosaki-u.ac.jp (Y.T.); shingoh@hirosaki-u.ac.jp (S.H.); goodwin@hirosaki-u.ac.jp (T.K.); coyama@hirosaki-u.ac.jp (C.O.); 2 Diagnostics Research Laboratories, Wako Pure Chemical Industries, Hyogo 661-0963, Japan; kurosawa.tatsuo@wako-chem.co.jp (T.K.); nakamura.kenji@wako-chem.co.jp (K.N.); 3 Department of Advanced Transplant and Regenerative Medicine, Hirosaki University Graduate School of Medicine, Hirosaki 036-8562, Japan; bikkuri@opal.plala.or.jp; 4 Department of Urology, Akita University Graduate School of Medicine, Akita 010-8543, Japan; narishin@doc.med.akita-u.ac.jp (S.N.); thabuchi@doc.med.akita-u.ac.jp (T.H.); 5 Department of Urology, Tohoku University Graduate School of Medicine, Sendai 980-8574, Japan; mitsuzuka@uro.med.tohoku.ac.jp (K.M.); yarai@uro.med.tohoku.ac.jp (Y.A.); 6 Department of Surgery, McMaster University, Hamilton, ON L8S4L8, Canada; duiven@mcmaster.ca (W.D.); pinthusj@hhsc.ca (J.H.P.)

**Keywords:** prostate-specific antigen, α2,3-linked sialyl *N*-glycan, *Maackia amurensis* lectin (MAA) lectin, biomarker

## Abstract

The low specificity of the prostate-specific antigen (PSA) for early detection of prostate cancer (PCa) is a major issue worldwide. The aim of this study to examine whether the serum PCa-associated α2,3-linked sialyl *N*-glycan-carrying PSA (S2,3PSA) ratio measured by automated micro-total immunoassay systems (μTAS system) can be applied as a diagnostic marker of PCa. The μTAS system can utilize affinity-based separation involving noncovalent interaction between the immunocomplex of S2,3PSA and *Maackia amurensis* lectin to simultaneously determine concentrations of free PSA and S2,3PSA. To validate quantitative performance, both recombinant S2,3PSA and benign-associated α2,6-linked sialyl *N*-glycan-carrying PSA (S2,6PSA) purified from culture supernatant of PSA cDNA transiently-transfected Chinese hamster ovary (CHO)-K1 cells were used as standard protein. Between 2007 and 2016, fifty patients with biopsy-proven PCa were pair-matched for age and PSA levels, with the same number of benign prostatic hyperplasia (BPH) patients used to validate the diagnostic performance of serum S2,3PSA ratio. A recombinant S2,3PSA- and S2,6PSA-spiked sample was clearly discriminated by μTAS system. Limit of detection of S2,3PSA was 0.05 ng/mL and coefficient variation was less than 3.1%. The area under the curve (AUC) for detection of PCa for the S2,3PSA ratio (%S2,3PSA) with cutoff value 43.85% (AUC; 0.8340) was much superior to total PSA (AUC; 0.5062) using validation sample set. Although the present results are preliminary, the newly developed μTAS platform for measuring %S2,3PSA can achieve the required assay performance specifications for use in the practical and clinical setting and may improve the accuracy of PCa diagnosis. Additional validation studies are warranted.

## 1. Introduction

**Figure 1:**
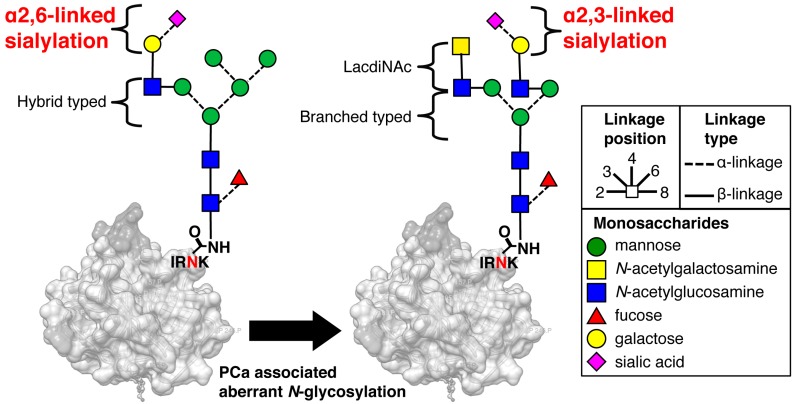
Prostate cancer-associated aberrant glycosylation of *N*-glycan on prostate-specific antigen (PSA). In normal PSA, the terminal sialic acids link to galactose residues with an α2,6 linkage whereas in prostate cancer (PCa)-associated PSA, the linkage between the terminal sialic acid and galactose residues is an α2,3 linkage [18]. Carbon linkage positions are denoted by the bond position drawn on each monosaccharide. Ile-Arg-Asn-Lys, (IRNK): *N*-glycosylation site of PSA.

Serum prostate-specific antigen (PSA) is widely used as a powerful biomarker for detecting prostate cancer (PCa) [1,2]. However, PSA-based PCa screening has resulted in over-diagnosis, leading to unnecessary prostate biopsies and overtreatment of indolent cancer [3,4,5]. Therefore, novel, more specific screening methods are urgently needed, especially for young healthy men. Of the various molecular isoforms of PSA, proPSA is one of the most promising potential biomarkers [6,7,8,9,10]. A multi-biomarker test for identifying aggressive prostate cancer that combines total PSA, free PSA, intact PSA, and human kallikrein-2 has been developed [11,12]. In contrast, we focused on the cancer-associated glycan alterations that have frequently been observed during carcinogenesis [13,14]. More importantly, some glycans, for example α-fetoprotein [15] and human chorionic gonadotropin [16], have been found to have specific cancer-associated carbohydrate alterations compared with their normal counterparts. The glycoprotein PSA has an *N*-glycosylation site on its 45th amino acid from the N-terminus. In a prior study, we cleaved a PSA-specific sequence (Ile-Arg-Asn-Lys, IRNK) that includes the glycosylated-Asn (N) and performed an intensive structural analysis of the glycan profile of PSA using matrix-assisted laser desorption/ionization time-of-flight (MALDI-TOF) mass spectrometry [17]. This resulted in our identification of a PCa-associated aberrant glycosylation of PSA, which produces α2,3-linked sialyl *N*-glycan that is readily observed on free PSA (S2,3PSA), whereas α2,6-linked sialyl *N*-glycan on free PSA (S2,6PSA) is exclusive to benign prostatic hyperplasia (BPH) [18] (Figure 1). 

We have also previously developed a magnetic bead-based S2,3PSA assay (Luminex method) that more accurately diagnoses early PCa than the conventional PSA test [19]. However, this method cannot measure benign S2,6PSA and does not have enough quantitative capability for clinical application. With the aim of overcoming these issues, we investigated using microfluidic technology, also referred to as micro-total analysis systems (μTAS system) [20,21,22,23], to quantitate both S2,6PSA and S2,3PSA in patient serum samples with a one-time measurement. μTAS immunoassays incorporating an automated platform can achieve the required assay performance specifications in clinical laboratories more efficiently than existing methodologies. In this study, we developed an automated μTAS system for measuring serum S2,3PSA ratio (%S2,3PSA) in clinical settings.

## 2. Results

### 2.1. Preparation of FLAG-Tag-Fused Recombinat S2,3 and S2,6 PSA in Chinese Hamster Ovary (CHO) Cells

**Figure 2:**
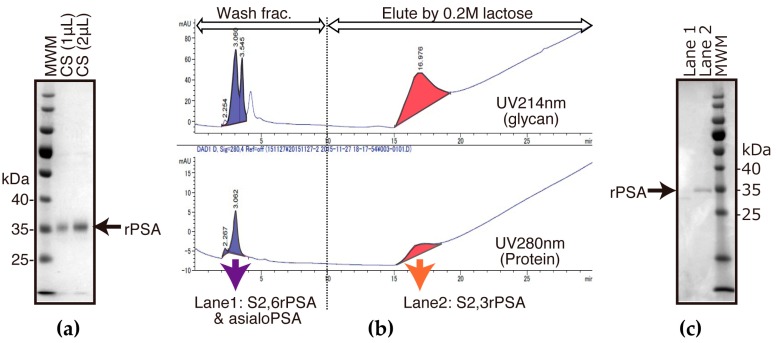

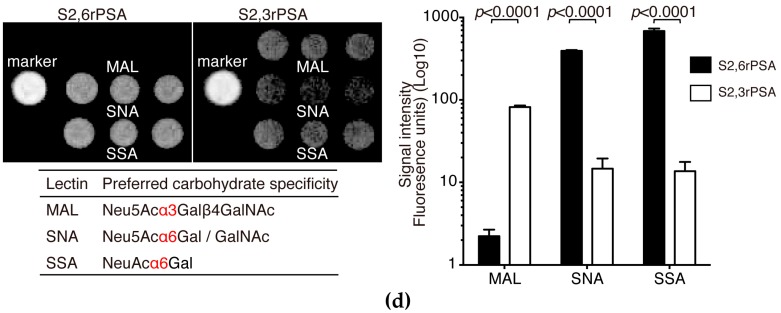
Preparation of α2,3-linked sialyl *N*-glycan-carrying PSA (S2,3PSA) and α2,6-linked sialyl *N*-glycan-carrying PSA (S2,6PSA) in Chinese hamster ovary (CHO)-K1 cells transfected with PSA–FLAG. CHO-K1 cells were transfected with FLAG-tag-fused human PSA cDNA (PSA–FLAG). (**a**) Culture supernatant (CS) with serum free media were blotted onto polyvinylidene fluoride (PVDF) membrane and probed with anti-FLAG antibodies; (**b**) Chromatograms obtained using *Agrocybe cylindracea* (ACG) lectin column chromatography for α2,3-linked sialyl *N*-glycan-carrying recombinant PSA (S2,3rPSA) collected from 0.2 M lactose eluted fraction and other glyco-isoform free PSA containing α2,6-linked sialyl *N*-glycan (S2,6rPSA) or asialo-type *N*-glycan collected from washed fraction. S2,3rPSA and S2,6PSA was further purified by gel filtration column chromatography; (**c**) Washed fraction (lane 1) and 0.2 M lactose-eluted fraction (lane 2) (indicating chromatogram) (**b**) were blotted onto PVDF membrane and probed with anti-FLAG antibodies; (**d**) Lectin array profiling of S2,3rPSA and S2,6rPSA proteins. Purified S2,3rPSA and S2,6rPSA was applied to the lectin array, including triplicate spots. The glass slides were scanned using a GlycoStationReader1200 (Glycotechnica, Sapporo, Japan). MWM: molecular weight markers; MAL: *Mackkia amurensis* lectin; SNA: *Sambucus Nigra* lectin; SSA: *Sambucus sieboldiana* lectin.

To construct S2,3PSA and S2,6PSA recombinant standard protein, FLAG-tag-fused human PSA cDNA (PSA–FLAG) was transiently transfected into CHO cells. Results of immunoblotting of PSA–FLAG-transfected CHO culture supernatant are shown in Figure 2a. Using an *Agrocybe cylindracea* (ACG) lectin affinity column and gel filtration column chromatography, S2,3PSA and S2,6PSA containing asialo-type *N*-glycan carrying recombinant PSA standard protein was purified from culture supernatants of human PSA expressed by CHO cells (Figure 2b,c). Lectin array analysis then revealed the putative glycan structure of purified S2,3 and S2,6PSA recombinant standard (Figure 2d). Signal intensity of recombinant S2,3PSA against *Mackkia amurensis* lectin (MAL) that preferentially bound the sialic acid α2,3-linked galactose structure was much higher than that of recombinant S2,6PSA. Signal intensity of recombinant S2,3PSA against *Sambucus Nigra* lectin (SNA) and *Sambucus Sieboldiana* lectin (SSA) that preferentially bound the sialic acid α2,6-linked galactose structure was much lower than that of recombinant S2,6PSA. These results suggest that the culture supernatant of PSA–FLAG-transfected CHO cells contained both α2,3- and α2,6-linked sialyl *N*-glycan-carrying recombinant PSA and could be utilized as a standard protein of μTAS system measuring S2,3PSA ratio (%S2,3PSA).

### 2.2. S2,6PSA and S2,3PSA Separation in a Microfluidic Channel Filled with Electrophoresis Leading Buffer Containing Maackia Amurensis Lectin

**Figure 3:**
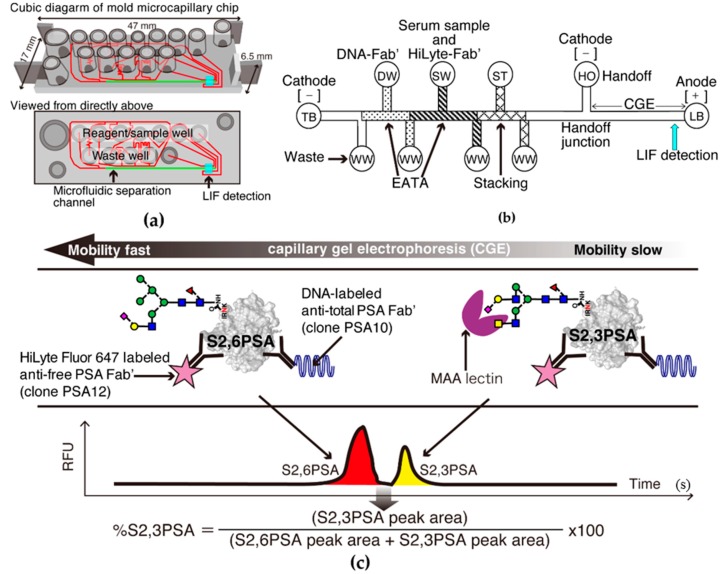
%S2,3PSA test using micro-total immunoassay systems (μTAS)-based microcapillary electrophoresis. (**a**) Molded plastic chip with top wells for reagents and precision microchannels on the bottom. Red line indicates microchannels. Highlight green line indicates microcapillary separation channel. Fluorescence signals are detected through the thin film closing the channels on the bottom of the chip; (**b**) Schematic diagram of a chip showing the electrokinetic analyte transport assay (EATA) method. Waste wells (WWs), trailing buffer (TB) well, leading buffer (LB), handoff (HO), DNA–labeled anti-total PSA Fab′ (DNA–Fab′) (DW), serum sample and HiLyte Fluor 647–labeled anti-free PSA Fab′ (HiLyte–Fab′) mixture (SW), and stacking buffer (ST) wells are shown. Vacuum applied to the WWs loads the reagents and sample-to-chip channel segments and voltage applied between cathode and anode mixes the sample and reagents for the binding reaction. Switching the voltage from the TB well to the HO well switches from isotachophoresis (ITP) stacking mode to capillary gel electrophoresis (CGE) mode. Separation of S2,3PSA from other glyco-isoform of free PSA occurs in the *Mackkia amurensis* lectin (MAA)-filled CGE separation channel prior to laser-induced fluorescence (LIF) detection. (**c**) Typical electropherogram of S2,6PSA and S2,3PSA peak separation. Fluorescent markers have been coelectrophoresed to identify the PSA protein peak positions (data not shown). RFU: relative fluorescence units.

Simultaneous quantitative measurement of S2,6PSA and S2,3PSA was achieved by performing microchip capillary electrophoresis and liquid-phase binding assay (LBA) on a μTAS Wako i30 auto analyzer with microfluidic chip IO6 (Wako Pure Chemical Industries, Hyogo, Japan) for electrokinetic analyte transport assay (EATA) (Figure 3a–c). Total assay time of μTAS system measuring %S2,3PSA ratio was 9 min, substantially shorter than the previously developed Luminex method (4 h). The reaction time during transport of DNA conjugate and bound free PSA analyte through the chip channels is short (25–50 s), and the binding kinetics are greatly increased by isotachophoresis (ITP) stacking and concentration of the reactants (Figure 3b) [24,25]. Figure 3c shows a typical electropherogram of S2,6PSA and S2,3PSA in a microfluidic channel separated by lectin affinity electrophoresis. Because S2,3PSA is reactive to *Mackkia amurensis* lectin (MAA), electrophoretic migration of the sandwich complex of DNA–labeled anti-total PSA Fab′ (DNA–Fab′), S2,3PSA, and HiLyte Fluor 647–labeled anti-free PSA Fab′ (HiLyte–Fab′) in the separation channel is slower than that of the immunocomplex of DNA–Fab′, S2,6PSA, and HiLyte–Fab′.

**Figure 4:**
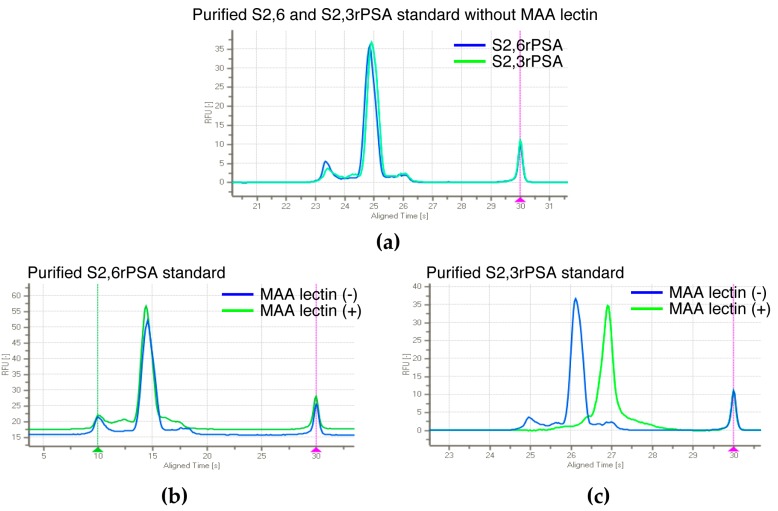
Capture efficiency of applied S2,3PSA standard in μTAS assay. (**a**) Electropherogram of S2,6rPSA and S2,3rPSA without MAA lectin. S2,6rPSA (**b**) and S2,3rPSA (**c**) standard were applied to the detection of peak separation respectively in a microfluidic channel filled with leading buffer (LB) containing MAA lectin (+) or not (−). The capture efficiency was evaluated by the peak mobility shift based on the specific interaction with lectin reactivity.

When MAA was eliminated from leading buffer (LB) in the separation channel, both S2,6PSA and S2,3PSA immunocomplex co-migrated to the same position (Figure 4a). Furthermore, using affinity-based separation with MAA lectin against aberrant glycosylation, we also confirmed a capture efficiency of applied S2,3rPSA standard of almost 100% in this assay (Figure 4b,c).

### 2.3. Assay Linearity and Sensitivity of %S2,3PSA Test by μTAS System

**Figure 5:**
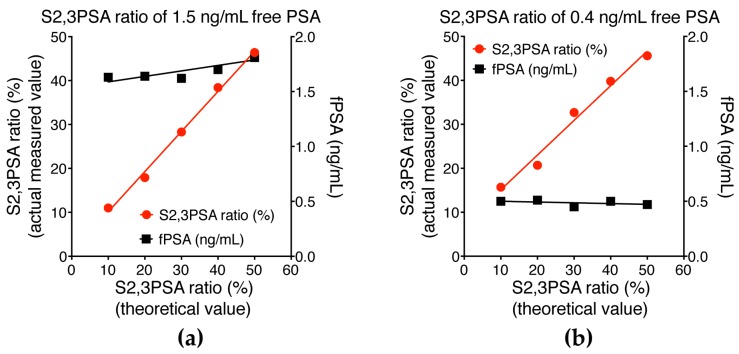

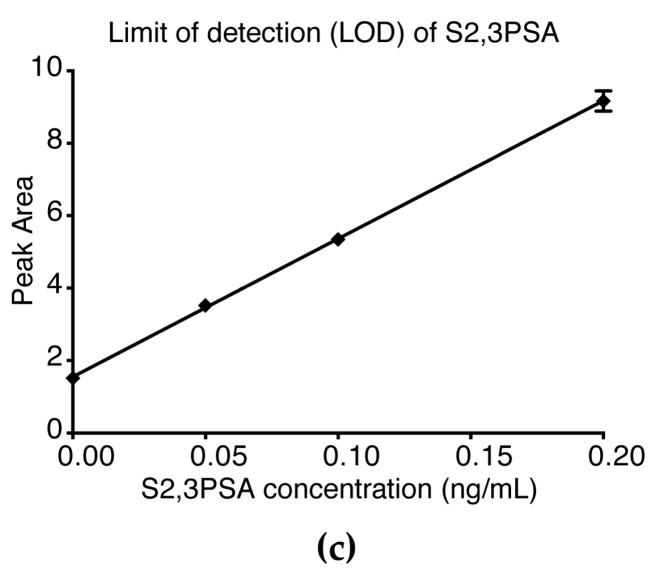
Assay linearity for measurement of S2,3PSA ratio (%S2,3PSA) and limit of detection of S2,3PSA measurement. (**a**) Sample was a prepared sample, the original sample having an PSA concentration of 1.5 ng/mL and %S2,3PSA of 50.0%; (**b**) Sample was prepared by spiking with PSA (0.4 ng/mL) and %S2,3PSA at 50%. The two samples were serially diluted by control PSA containing S2,6 sialylation or asialo at the same concentration. PSA concentrations and %S2,3PSA were determined by the μTAS system; (**c**) The limit of detection (LOD) was 0.05 ng/mL, this level showing no overlap between the 2 standard deviation (SD) ranges for S2,3PSA concentration and negative control.

To demonstrate that our assay is robust for determining %S2,3PSA, recombinant standard samples were serially diluted with control PSA protein and %S2,3PSA determined at a constant concentration of free PSA. As shown in Figure 5a,b, there was a linear relationship between percentage of S2,3PSA and fluorescence intensities in the two prepared samples tested. The assay’s sensitivity was determined by testing samples of buffer spiked with serially diluted S2,3PSA with the means ± 2 standard deviations (SD) being calculated for five replicates. As shown in Figure 5c, 0.05 ng/mL S2,3PSA was clearly detectable over the zero sample, there being no overlap of the 2SD range with zero. The reproducibility of the peak area detection for the 0.05 ng/mL level was within 15% coefficient of variation (CV) for %S2,3PSA, indicating that the limits of detection of quantitation of the assay are 0.05 ng/mL.

### 2.4. Assay Reproducibility of %S2,3PSA Test Using the μTAS System

Assay reproducibility was examined by measuring two samples at each concentration with total of free PSA and %S2,3PSA, using 10 replicate measurements of each sample. We confirmed the assay’s very good reproducibility. The CV was calculated to within 3% for free PSA and 4% for %S2,3PSA for all ranges tested (Table 1).

### 2.5. Validation of %S2,3PSA Test

**Figure 6:**
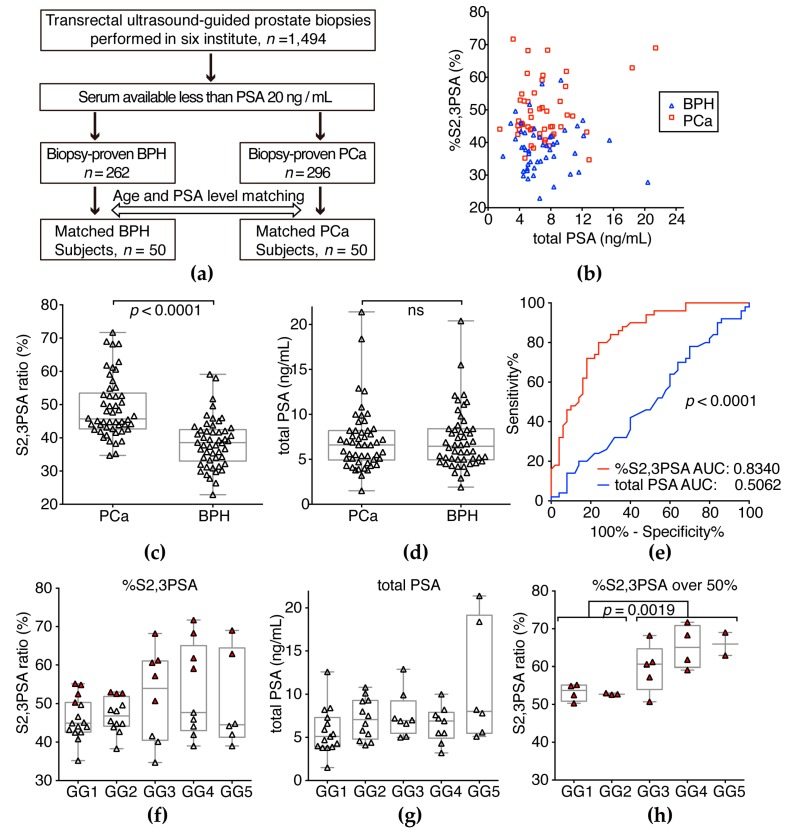
Serum %S2,3PSA and total PSA level in the validation sample set. (**a**) Fifty patients with PCa were pair-matched for age and PSA level with the same number of patients with BPH; (**b**) Correlations between %S2,3PSA and total PSA value in patient serum samples; (**c**) Serum %S2,3PSA was significantly higher in patients with PCa than BPH (*p* < 0.0001). The grand mean represents the overall mean of the Y variable; (**d**) Serum total PSA was not significantly different between PCa and BPH (**e**) Receiver operating characteristics (ROC) curve analysis for detection of PCa showed that the areas under the curve (AUC) for %S2,3PSA and conventional total PSA tests were 0.8340 and 0.5062, respectively; (**f**) %S2,3PSA among PCa patients classified by the Pbx GG; (**g**) total PSA level of PCa patients classified by the Pbx GG. Red triangle indicates %S2,3PSA over 50%; (**h**) Comparison between Pbx GG ≤ 2 and Pbx GG ≥ 3 patients that had an %S2,3PSA ratio over 50% (*p* = 0.0019).

Relevant clinical details of patients from whom samples were obtained are shown in Table 2. In this study, age and PSA level of 100 matched serum samples were assessed, comprising 50 from patients with PCa and 50 from BPH cases (Table 2, Figure 6a). Figure 6c shows that %S2,3PSA was significantly higher in patients with PCa than in patients with BPH (*p* < 0.0001). Total PSA level was not significantly different between the groups (Figure 6d). Receiver operating characteristic curve analyses were then used to compare the diagnostic potential of total PSA and %S2,3PSA (Figure 6e). The area under the curve (AUC) showed that results of conventional PSA testing did not differ between patients with BPH and PCa (AUC 0.5062, 95% CI 0.3922–0.6202), whereas there was a good separation for %S2,3PSA (AUC 0.8340, 95% CI 0.7555–0.9125, *p* < 0.0001) without any correlation between each assay (Figure 6b). The optimum cutoff point giving high specificity (72.0%) at 80% sensitivity was determined to be 42.20% of %S2,3PSA, with positive and negative predictive values of 75.5% and 78.7%, respectively, and much superior total PSA specificity (14.0%) at 80% sensitivity was determined to be 4.45 ng/mL of total PSA, with positive and negative predictive values of 48.2% and 41.2%, respectively. In this validation sample set, 54% (*n* = 27/50) of patients with PCa were found to be in the low Grade Group (GG) [26], with GG 1 (Gleason Score, GS 3 + 3) and GG 2 (GS 3 + 4) tumors. In addition, 46% (*n* = 23/50) of patients with PCa were found to have a greater than GG 3 (GS 4 + 3) tumor by prostate biopsy (Pbx) (Table 2). Figure 6g,f shows the total PSA and %S2,3PSA ratio of PCa patients classified by the prostate biopsy grade group (Pbx GG). The %S2,3PSA and total PSA level showed no significant difference between Pbx GG ≤ 2 and Pbx GG ≥ 3. In the case of greater than 50% S2,3PSA ratio, %S2,3PSA of Pbx GG ≥ 3 patients (range 50.7%–71.7%)(47%, *n* = 11/23) was significantly higher than that of Pbx GG ≤ 2 patients (range 50.3%–52.6%) (26%, *n* = 7/27) (*p* = 0.0019).

## 3. Discussion

The use of aberrant PSA glycosylation for early detection of prostate cancer has been reported [27,28,29,30]. In particular, we have previously identified that the terminal *N*-glycan structure of PSA from patients with PCa is rich in sialic acid α2,3-linked to the galactose residue, whereas the terminal *N*-glycan structures of PSA from seminal plasma are exclusively α2,6-linked [17]. We have also developed a magnetic bead-based S2,3PSA assay (Luminex method, [19]) that more accurately diagnoses early PCa than conventional PSA testing; however, this method is not sufficiently versatile and does not have enough quantitative capability for clinical application. To overcome these issues, we here developed an automated microcapillary electrophoresis-based immunoassay system (μTAS system) to be able to measure serum %S2,3PSA in clinical settings.

In this study, we developed a μTAS system based on the principles of EATA and LBA, precision-injection molded microfluidic plastic chips, and instrumentation optimized for running microfluidic chips and demonstrated that this EATA method can utilize affinity-based separation involving noncovalent interaction between the immunocomplex of α2,3-linked sialylated PSA glyco-isoform and MAA to simultaneously determine concentrations of total free PSA and its glyco-isoform, S2,3PSA. We have previously demonstrated that the migration speed of an immunocomplex of DNA–Fab in a microfluidic channel can be controlled by changing the length of the DNA fragment [31]; capillary gel electrophoresis (CGE) is also known to be capable of separating molecules on the basis of size and charge.

In this study, we established an assay standard with recombinant S2,3PSA protein expressed by CHO cells and a standard curve for the quantitative measurement of %S2,3PSA according to assay linearity (Figure 5a,b). We also confirmed a capture efficiency of applied S2,3PSA standard of almost 100% in this assay (Figure 4b,c). By using specific lectin MAA to recognize α2,3-linked sialylation directly, the calculating analyte percentage excluded an unspecified factor contributed by different glyco-isoforms like asialo-type glycan as compared to the indirect assay with other lectin-based approaches [32]. These observations suggest that our assay strategy allows the reliable determination of the percentage ratio of S2,3PSA without loss of capturing the specific PSA glyco-isoform.

In terms of assay performance, the sensitivity for detection of S2,3PSA was 0.05 ng/mL using the μTAS immunoassay system. As noted in previous studies of EATA methods [33], up to 140-fold concentrations of sample and reagent mixtures can be achieved by ITP stacking, greatly enhancing assay sensitivity and binding kinetics. The newly developed μTAS system has higher analytical sensitivity than our previous methods [18,19] and %S2,3PSA can be determined at lower free PSA concentrations. This higher analytical sensitivity enables low CVs (<4%) for a precise percentage ratio of S2,3PSA and should improve the clinical utility of %S2,3PSA in patients with small tumors. Clinical studies employing the μTAS system are currently underway; the results will be published separately.

In addition to its high sensitivity, the μTAS in %S2,3PSA assay also shortens time to first result from the 4 h required for our previous assay using the Luminex method, [19] to less than 10 min. We achieved this improvement by combining the LBA principle and microchip capillary electrophoresis. Because LBA does not rely on diffusion of antigen to antibody immobilized on the surfaces of a solid phase, the immunoreaction is quick and stoichiometric. Furthermore, in the on-chip system, electrophoresis can be performed quickly by applying higher voltage under better thermal control; the principle of EATA results in a concentration of reactants that shortens binding and separation times [25,33].

In the present study, a novel assay system for measuring %S2,3PSA with a μTAS system discriminated patients with PCa from patients with BPH with 72.0% specificity at 80.0% sensitivity with a α2,3-sialic acid percentage cut-off of 42.20%, as indicated by an AUC of 0.8340 which was significantly higher than that for conventional PSA testing (0.5062, *p* < 0.0001 Figure 6e). According to some reports [10,11,12], combination assay containing multiple biomarkers such as the 4K test or Prostate Health Index would be applicable for improving diagnostic accuracy. In contrast, since %S2,3PSA test is a single biomarker assay based on the measurement of aberrant glycosylation, this simple assay strategy would achieve easy-to-use clinical application with a higher accuracy of diagnosis of PCa. Additionally, %S2,3PSA of Pbx GG ≥ 3 patients (range 50.7%–71.7%) was significantly higher than that of Pbx GG ≤ 2 patients (range 50.3%–52.6%) in the case of an over-50% S2,3PSA ratio (*p* = 0.0019) (Figure 6h). Although sample size was small, this result suggests that an over-50% S2,3PSA ratio holds the promise to discriminate between GG 2 and GG 3 tumors and may be used as a predictor for a prostate biopsy to discriminate between non-aggressive and aggressive tumors in the active surveillance program. To address this issue, we would need larger cohort study in the future.

## 4. Materials and Methods

### 4.1. Immunoassay Reagents

DNA (245-bp) and HiLyte Fluor 647 dye (AnaSpec, San Jose, CA, USA) were coupled to Fab′ prepared from anti-total PSA and anti-free PSA monoclonal antibodies (clones PSA10 and PSA12, respectively) that had been selected from our panel of mouse immunoglobulin G antibodies. These antibodies were digested with pepsin, followed by reduction of F(ab′)2 using 50 mM 2-aminoethanethiol to form Fab′. Each Fab′ was purified by Diol 200 gel filtration column chromatography (Wako Pure Chemical Industries, Osaka, Japan).

DNA-labeled anti-total PSA Fab′ (clone PSA10) was prepared as follows, a 245-bp DNA fragment was amplified by polymerase chain reaction using lambda DNA as a template and 5′ amine-modified primer as a forward primer to provide the amino group needed to couple the DNA fragment to anti-total PSA (clone PSA10) Fab′ antibody via bifunctional linker. Gene Taq polymerase (Wako Pure Chemical Industries) was used as polymerase. The DNA fragment was purified by using Diol-200 gel filtration and DEAE ion exchange chromatography on a DEAE-P5 W column (Tosoh, Tokyo, Japan). Next, 10 μM of the 245-bp DNA fragment was reacted with 10 mM *N*-(6-maleimidocaproyloxy)succinimide (EMCS) linker (Wako Pure Chemical Industries), a bifunctional linker having both maleimide and succinimide groups, at 37 °C for 30 min in 50 mM phosphate-buffered saline (PBS, pH 7.5). The linker-modified, 245-bp DNA fragment was purified by Diol-200 gel filtration, concentrated to 10 μM, and reacted with 500 μM PSA10 Fab′ at 4 °C for 3 h in 50 mM PBS (pH 6.5). To remove DNA-coupled Fab′ that had more than one DNA fragment, the conjugate was further purified by both Diol-200 gel filtration and DEAE column chromatography.

HiLyte Fluor 647 labeled anti-free PSA Fab′ (clone PSA12) was prepared as follows, anti-free PSA monoclonal antibody (clone PSA12) Fab′ was reacted with 1 mM HiLyte Fluor 647 C2 maleimide (Wako Pure Chemical Industries) at 4 °C for 2 h in 50 mM PBS (pH 7.5), after which 1 mM *N*-ethylmaleimide was added to the reaction mixture to block free thiol groups on the Fab′ molecule after the labeling reaction. Excess unreacted HiLyte Fluor 647 C2 maleimide was removed by Diol-200 gel filtration.

Electrophoresis leading buffer (LB) and trailing buffer (TB) for isotachophoresis (ITP) and capillary electrophoresis (CE) were formulated by adding nonionic surfactants to block the plastic chip surface and facilitate chip filling. The composition of the LB was 4.5% (*w*/*v*) polyethylene glycol (PEG8000), 3% (*w*/*v*) glycerol, 75 mM Tris–HCl (pH 7.5), 10 mM NaCl, 6.0% (*w*/*v*) dextran sulfate, 0.01% bovine serum albumin (BSA), and 4 mg/mL *Maackia amurensis* lectin (MAA). The composition of the sample buffer (SB) was 5.0% (*w*/*v*) PEG20000, 3% (*w*/*v*) glycerol, 75 mM Tris–HCl (pH 7.5), 150 mM NaCl, 0.01% BSA, and 10 mM 2-(*N*-morpholino)ethanesulfonic acid (MES). The TB consisted of 2.0% (*w*/*v*) PEG20000, 3% (*w*/*v*) glycerol, 75 mM Tris, 0.01% BSA, and 125 mM Hepes. The stacking buffer (ST) was composed of 2.0% (*w*/*v*) PEG20000, 3% (*w*/*v*) glycerol, 75 mM Tris–HCl (pH 7.5), and 0.01% BSA.

### 4.2. Microfluidic Electrophoresis Assay

Simultaneous quantitative measurement of S2,6PSA and S2,3PSA was achieved by performing microchip capillary electrophoresis and liquid-phase binding assay (LBA) on a μTAS Wako i30 auto analyzer with microfluidic chip IO6 (Figure 2a,b; Wako Pure Chemical Industries) for electrokinetic analyte transport assay (EATA). The details of the EATA method have been described by us previously [24] and are shown in Figure 2b. In brief, HyLite–Fab′ was mixed with the serum sample in SB and the resulting mixture loaded into a sample well. DNA–Fab′, the LB containing MAA, and the TB were also loaded into their designated wells, and the ST and focusing dye solutions into the ST well and focusing dye solution well, respectively. After all buffers and sample had been loaded, positive pressure (+20 psi) was applied to all wells to equilibrate all zones. Next, an electrical field was applied from the cathode to the anode to initiate ITP stacking of the DNA–Fab′. The stacked DNA–Fab′ migrated from the DNA–Fab′ zone into the sample + HiLyte–Fab′ zone to initiate formation of the sandwich immunocomplex of DNA–Fab′, PSA, and HiLyte–Fab′. The resulting immunocomplex was then further transported to the separation zone by ITP. During the ITP step, unreacted HiLyte–Fab′ was left behind in the serum sample and HiLyte–Fab′ zone. When the boundary of TB and LB reached the handoff junction, the electrical field was automatically switched from cathode–anode wells to handoff–anode wells and capillary gel electrophoresis (CGE) started to both separate the remaining noise components from the immunocomplex and S2,3PSA from S2,6PSA by affinity electrophoresis in an MAA-containing separation gel. Fluorescence signals from laser-induced fluorescence (LIF) detection were analyzed by software developed to use internal fluorescent markers to align and identify the peaks for S2,6PSA and S2,3PSA. The analyte peaks were integrated for peak area, which was then used to quantitate their ratio, as shown in Figure 2c.

### 4.3. Prostate Biopsy and Serum Samples

Between June 2007 and June 2016, 1494 transrectal ultrasound-guided prostate biopsies were performed in response to detection in regional PCa screening programs of PSA concentrations of ≥4.0 ng/mL or palpable prostate nodules in Hirosaki University Hospital, Akita University hospital, Tohoku University Hospital and McMaster University (Juravinski Hospital, Hamilton, ON, Canada). Serum samples were obtained from all patients at the time of biopsy and stored at −80 °C until use. The final diagnoses were established by histopathological examination of prostate biopsies. The grade group of prostate biopsy specimens were evaluated according to the International Society of Urological Pathology (ISUP) guidelines [26]. This study was performed in accordance with the ethical standards of the Declaration of Helsinki and was approved by the Ethics Committees of all participating institute including Hirosaki University Graduate School of Medicine (“The Study about Carbohydrate Structure Change in Urological Disease”; approval number: 2014–195). Informed consent was obtained from all patients. Table 2 shows relevant clinical characteristics of the study subjects. To evaluate diagnostic performance of serum S2,3PSA ratio, fifty patients with biopsy-proven PCa were pair-matched for age and PSA level with the same number of BPH patients selected from our serum bank.

### 4.4. Forced Expression of FLAG-Tag-Fused S2,3 and S2,6PSA in Chinese Hamster Ovary (CHO)-K1 Cells

CHO-K1 cells were obtained from the American Type Culture Collection and grown in Ham’s F12 Nutrient Mixture medium supplemented with penicillin, streptomycin, and 10% fetal bovine serum at 37 °C with 5% CO_2_. FLAG-tag (N-DYKDDDDK-C)-fused human PSA (kallikrein-3, KLK3) cDNA was amplified from RNA isolated from the prostate of a patient with benign prostatic hyperplasia using the primers hPSA-F1 5′-CCCAAGCTTACCACCTGCAC-3′ and hPSA-FLAG-Xho-R1 5′-TTTCTCGAGCTACTTGTCATCGTCGTCCTTGTAATCAGCGGGGTTGGCCACGATGGT-3′ and subcloned into the PCaG-Neo vector (Wako Pure Chemical Industries). The PSA–FLAG vector was then transiently transfected into CHO-K1 cells. After transfection, recombinant PSA was purified using a FLAG-tag system (Sigma, St. Louis, MO, USA) from serum free media. S2,3PSA and S2,6PSA containing asialo-type standard protein was purified utilizing an ACG lectin column and gel filtration column chromatography.

### 4.5. Lectin Microarray

One hundred microliters of purified S2,6PSA and S2,3PSA (31.25–2000 ng/mL) were applied to a lectin array (LecChipver1.0; GlycoTechnica, Sapporo, Japan), including triplicate spots of lectins in each of seven divided incubation baths on glass slides [34]. After incubation at 20 °C for 17 h, the reaction solution was discarded and the glass slides scanned using a GlycoStation Reader1200 (GlycoTechnica). Abbreviation of lectins are as follows: MAL, *maackia amurensis* lectin; SNA, *sambucus nigra* lectin; and SSA, *Sambucus sieboldiana* lectin.

### 4.6. Statistical Analysis

All calculations for clinical data were performed in the SPSS software, ver. 21.0 (SPSS, Inc., Chicago, IL, USA) and in GraphPad Prism 6.03 (GraphPad Software, San Diego, CA, USA). Intergroup differences were statistically analyzed by a Student’s *t*-test for normally distributed variables or by the Mann–Whitney *U*-test for non-normally distributed models. Data with *p* < 0.05 were considered significant. Receiver operating characteristics (ROC) curves were developed using the library “rms” in R (available on: http://www.r-project.org/) [25] and the statistical difference of AUCs were calculated by the same program.

## 5. Conclusions

In conclusion, although the present results are preliminary, they suggest that the newly developed serum %S2,3PSA test may have superior diagnostic accuracy to currently available tests. Additionally, we believe that the %S2,3PSA assay with μTAS system has great potential for further application in the clinical laboratory when rapid and quantitative testing is required. Larger-scale studies are warranted to confirm these findings.

## Figures and Tables

**Table 1:** Assay reproducibility of S2,3PSA test.

Sample Number	1.0 ng/mL Free PSA		5.0 ng/mL Free PSA	
	Free PSA	%S2,3PSA	Free PSA	%S2,3PSA
1	1.04	50.5	5.06	38.1
2	0.99	50.7	5.08	38.1
3	1.04	49.4	5.00	38.3
4	1.05	50.1	5.07	37.9
5	1.04	47.7	5.05	37.7
6	1.03	50.4	5.24	37.7
7	1.13	50.3	5.13	38.3
8	1.10	46.8	5.01	37.9
9	1.05	47.1	5.04	38.1
10	1.01	48.5	5.21	37.9
Ave. ^1^	1.04	49.1	5.09	38.0
SD ^2^	0.03	1.51	0.08	0.21
CV ^3^	2.8%	3.1%	1.6%	0.6%

^1^ Ave.: average; ^2^ SD, standard deviation; ^3^ CV: coefficient variation.

**Table 2:** Age and total PSA BPH and PCa patients in the validation study.

Characteristics	BPH ^a^	PCa ^b^	*p* (^a^ vs. ^b^)
*n* = 100	50	50	
Age, median (range)	66.5 (51–85)	67 (51–86)	ns ^1^
PSA ^2^, ng/mL, median (range)	6.45 (1.9–20.4)	6.6 (1.5–21.4)	ns ^1^
%S2,3PSA, median (range)	38.55 (22.9–59.1)	45.70 (34.7–71.7)	<0.0001
pbx GG ^3^		n (%)	
GG 1		15 (30%)	
GG 2		12 (24%)	
GG 3		8 (16%)	
GG 4		9 (18%)	
GG 5		6 (12%)	

^1^ no significant difference; ^2^ total PSA; ^3^ pbx GG: prostate biopsy grade group; ^a^ benign prostatic hyperplasia; ^b^ prostate cancer.

## Abbreviations

μTAS	micro-total analysis system
MAA	*maackia amurensis* lectin
EATA	electrokinetic analyte transport assay
LBA	liquid-phase binding assay
C.V.	coefficient of variation
ITP	isotachophoresis
LIF	laser-induced fluorescence
LB	leading buffer
TB	trailing buffer
CE	capillary electrophoresis
SB	sample buffer
ST	stacking buffer
CGE	capillary gel electrophoresis
LOD	limit of detection
PSA	prostate-specific antigen
PCa	prostate cancer
BPH	benign prostatic hyperplasia
Pbx GG	prostate biopsy grade group
LacdiNAc	GalNAcβ1-4GlcNAc-
Gal	galactose
Man	mannose
Fuc	fucose
Sia	sialic acid
GalNAc	*N*-acetylgalactosamine
GlcNAc	*N*-acetylglucosamine
